# Evaluation of β-blocker therapy for long-term outcomes in patients with low ejection fraction after cardiac surgery

**DOI:** 10.1186/s12872-020-01651-6

**Published:** 2020-08-20

**Authors:** Fu-Dong Fan, Hai-Tao Zhang, Tuo Pan, Xin-Long Tang, Dong-Jin Wang

**Affiliations:** 1 Department of Cardio-Thoracic Surgery, Nanjing Drum Tower Hospital, The Affiliated Hospital of Nanjing University Medical School, Number 321 Zhongshan Road, Nanjing, 210008 Jiangsu China; 2 Department of Cardio-Thoracic Surgery, Nanjing Drum Tower Hospital, Peking Union Medical College, Chinese Academy of Medical Sciences, Graduate School of Peking Union Medical College, Beijing, 100010 China

**Keywords:** Cardiac surgery, β-Blocker, Long-term survival

## Abstract

**Background:**

Preoperative low left ventricular ejection fraction (LVEF) has been reported as an independent risk factor for in-hospital mortality. However, there were few studies evaluating the long-term mortality in these patients. We, therefore, conducted this study to investigate long-term outcomes of surgery on patients with LVEF≤35% undergoing a broad range of cardiac procedures.

**Methods:**

We performed a retrospective cohort study in 510 patients from January 1, 2007 to September 1, 2019. These patients were divided into survival group (*n* = 386) and non-survival group (*n* = 124). The multivariate Cox analysis was used to estimate the risk factors for survival. In Cox analysis, β-blockers were indicated to be associated with long-term mortality. To further address bias, we derived a propensity score predicting the function of β-blockers on survival, and matched 52 cases to 52 controls with similar risk profiles.

**Results:**

Patients were followed for a median period of 24 months (interquartile range: 11–44 months). Multivariate Cox regression analysis indicated that the non-survival group had higher weight, higher EuroSCORE, more smoking patients, longer time of cardiopulmonary bypass (CPB), more intra-aortic balloon pump (IABP) use, and more patients who always used β-blocker (HR: 2.056, 95%CI:1.236–3.420, *P* = 0.005) compared with survival group. After propensity matching, the group which always used β-blocker showed higher rate of all-cause death compare with the control group (61.54% vs 80.77%, *P* = 0.030).

**Conclusions:**

The risk factors for long-term survival were weight, EuroSCORE, smoking, CPB, IABP, always used β-blockers in patients with LVEF≤35%. The discharge prescription of β-blocker should be cautiously administrated in those patients.

## Background

Preoperative low left ventricular ejection fraction (LVEF) is common in patients undergoing cardiac surgery, especially those scheduled for mitral valve replacement, aortic valve replacement and coronary artery bypass grafting (CABG) [1–3]. These previous studies have shown that LVEF, typically observed in the course of the disease, was an independent risk factor for overall mortality as well as for sudden cardiac death [1–5]. However, Gambardella et al. reported that LVEF was not an independent predictor of adverse events in surgery of the descending thoraco-abdominal aorta [6]. Alternative treatment among patients with low LVEF is limited. Owing to unsatisfactory treatment, some studies suggested that these patients should receive heart transplantation [7–9]. However, the heart transplantation is also limited due to lack of organ donors [10].

A retrospective study reported that an early referral for mitral valvuloplasty would improve survival even in patients with low LEVF [3]. Many studies have shown that CABG has a good survival in patients with significant left ventricular systolic dysfunction [11–13]. A systematic review supports the concept that patients with low LEVF show a clear functional improvement after aortic valve replacement [1]. In other word, patients might benefit from these traditional cardiac procedures if they don’t have donated hearts. As known, patients with low LVEF are at a higher risk for postoperative mortality [1–5]. Therefore, early recognition of patients at risk for poor outcomes plays a pivotal role in the decision making process. Several post- and peri-operative variables have been purposed as predictors of mortality, including mitral valve surgery [2], renal failure [14], low cardiac output syndrome [15], and bleeding requiring reoperation [16], etc. Nevertheless, previous studies have primarily addressed early outcomes, with limited contemporary data regarding long-term mortality in patients with low LVEF undergoing a broad range of cardiac procedures. Likewise, there are few data assessing the discharge prescriptions that might identify patients who are at highest risk. Therefore, we retrospectively analyzed our experience with patients undergoing cardiac surgery to evaluate the influence of LVEF ≤35% on the long-term outcomes. Furthermore, unlike some other studies [4, 11–13, 16], we also included patients who had undergone a broad range of cardiac procedures, such as mitral valve surgery, aortic valve surgery, CABG and ventricular aneurysmectomy, etc.

## Methods

### Study design and settings

This study is a retrospective, observational, convenience sample study that conducted at Nanjing Drum Tower Hospital (Nanjing, Jiangsu Province, China). After receiving approval from the ethical committee of Nanjing Drum Tower Hospital, data from all patients with LVEF ≤35% who underwent cardiac surgery over a 10 years’ period were collected. From January 1, 2007 to September 1, 2019, all consecutive patients with integrated hospital medical records, nursing records, laboratory database records, and cardiac surgery database records and undergoing a broad range of cardiac procedures were included. Patients were excluded if they were < 18 years-old had physician-documented contraindications to β-blocker therapy [17], congenital heart disease, pregnant, any re-operations, angioplasty or thrombolytic therapy, extracorporeal membrane oxygenation (ECMO) or intra-aortic balloon pump (IABP) initiation before operation.

### Patient management and follow up

All surgical operations were performed through a median sternotomy. The cardiopulmonary bypass (CPB) was used in all patients. The ascending aorta was cannulated with a patient size-appropriate cannula. Venous cannulations were chosen with separate cannulas in the superior and inferior vena cava. The initial volume of the antegrade cold blood cardioplegia solution (4:1 cardioplegia solution to blood ratio) was twice the volume needed for the cessation of all cardiac electrical activity but never less than 1000 m. Cardiac arrest was maintained by the retrograde infusion of 300 mL of blood cardioplegia solution (8:1 cardioplegia solution to blood ratio) every 20 min. Occasionally, intermittent antegrade cold blood cardioplegia was used according to the surgeon’s preferences. Whenever possible, the internal thoracic artery was preferentially used for revascularization of the left anterior descending artery. Then, arterial conduits or saphenous vein grafts were used for complete revascularization. The CABG was preferentially performed if patients underwent CABG combined with valvular or other cardiac surgery. The standard surgical techniques were performed during CPB.

There were 684 patients who met the including and excluding criteria in study cohort. The follow-up ended on October 1, 2019. The data were recorded at the outpatient visit or by telephone interview. Patients were encouraged to return for a routine outpatient visit at 3 months, 6 months, 12 moths, 18 months and 24 months after hospital discharge. Researchers would visit patients when they did not have outpatient visits or telephone interviews. At each visit, patients were asked to read all of their current medications to the interviewer, including drug name, dose and schedule. In this cohort, patients were followed for a median period of 24 months (interquartile range:11–44 months), and 174 patients were lost to follow-up. Therefore, 510 patients who were not lost at follow up were included in statistic analysis and were divided into survival group (*n* = 386) and non-survival group (*n* = 124).

The clinical outcomes were ascertained after the 12-months observational interval that was used for determining β-blocker adherence. The primary outcome was all cause death. The secondary outcomes were included refractory heart failure, stroke, refractory arrhythmia, myocardial infarction, renal failure, respiratory failure. In multivariate analysis, the continuous variables were converted to binary variables by upper quartile (≥75th percentile). Type of cardiac surgery included CABG, aortic valve replacement/repair (AVR), mitral valve replacement/repair (MVR), AVR + MVR, David/Wheats/Bentall procedure, CABG + ventricular aneurysmectomy and CABG + valvular surgery. In the overall cardiac surgical population, CABG is the most frequently performed operation [18], and ischemic cardiomyopathy is the most frequent cause of heart failure associated to a reduced LVEF [19]. We, therefore, use CABG to act as a reference in multivariable analysis. The continuous renal replacement therapy (CRRT) was initiated in the presence of an increase in serum creatine of more than 100% or oliguria persist for more than 4 h despite medications (urine output ≤0.5 ml/kg/h). The intra-aortic balloon pump (IABP) was initiated and implemented with standard guidelines [20, 21]. In preoperative patients with LVEF < 35%, the median follow-up time is < 24 months in many previous studies [22–24]. Our median follow-up time is 24 months (IQR:11-44 months). The “long-term mortality” in our study may be acceptable.

### Statistical analysis

IBM SPSS statistical software was used (Statistics for Windows, version 25, IBM Corporation, Armonk, NY, USA). Normally distributed variables were expressed as mean ± standard deviation and compared using Student’s t-test. Nonparametric continuous variables were expressed as median (interquartile range (IQR) and compared using the Mann-Whitney U test. Continuous variables were determined to be normal in distribution by the Shapiro-Wilk test. Categorical data were equated using the chi-square test or Fisher’s exact test. The Kaplan-Meier method was used to analyze unadjusted survival, which was compared using the log-rank test. All covariates reaching a statistical significance (*P* ≤ 0.10) in univariate modeling were entered into a multivariable Cox regression model designed to assess the independent association of risk factors for survival. Collinearity diagnostics were performed using tolerance estimates for individual variables in a linear regression model.

In multivariable Cox analysis, β-blockers were indicated to be associated with long-term mortality. Because β-blockers administration was identified as a novel risk factor of survival following cardiac surgery, we further adjusted bias by using a propensity score. Therefore, with the help of this method, a comparison between patients always used β-blockers (β-Blocker group) and those who never or inconsistently used β-blockers (control group) with similar risk profiles was made possible [25]. Before matching, there were 24 patients with no β-blocker treatment. After matching, there was no patients with no β-blocker treatment in control group. In both groups, the follow-up was completed on October 1, 2019. However, the mortality of β-blocker group was higher than the mortality of control group. Therefore, β-blocker group has shorter follow-up length (median: 17, IQR: 8–41 months) compered with control group (median: 26 months, IQR 12–47 months). Propensity score 1-to-1 matching was utilized with the nearest neighbor algorithm without replacement and a 0.02 caliper setting. Age, New York Heart Association (NYHA) class, history of acute myocardial infarction (AMI), smoking, type of cardiac surgery, CPB time, intraoperative bleeding, drainage on the first postoperative day (POD1), mechanical ventilation (MV) time, EuroSCORE and always use ACEI/ARB during follow up were put into a Logistic regression model to estimate the propensity score. Following matching, standardized mean differences (SMD) were used to assess the degree of baseline variable balance. The SMD between matching pairs < 0.20 reflects an acceptable balance. All reported *P* values are two sides, and values of *P* < 0.05 were considered to indicate statistical significance.

## Results

A total of 510 patients were enrolled in this study. Amongst them, 386 patents were survived without any complications, while 124 patients died during follow-up. Therefore, the survival group had 386 patients, and the 124 patients were included in non-survival group. All of patients underwent CABG received on-pump procedures. Additionally, patients who postoperatively initiated IABP were implemented CABG+ ventricular aneurysmectomy (*n* = 4) and CABG + valvular surgery (*n* = 8). The baseline and demographic variables were quite different between survival group and non-survival group (Table 1; eTable 1 and eTable 2 in the online-only Data Supplement).

**Table 1:** Preoperative Baseline and Characteristics

Variable	Non-Survival (***n*** = 124)	Survival (***n*** = 386)	P value
Age (year)	57.08 ± 13.05	59.67 ± 11.71	0.038
Gender (male, %)	98, 79.03%	239, 61.92%	< 0.001
Weight (kg)	66.96 ± 11.89	62.81 ± 11.85	0.001
NYHA class (n, %)			0.001
I	5, 4.04%	9, 2.33%	
II	55, 44.35%	109, 28.24%	
III	55, 44.35%	228, 59.07%	
IV	9, 7.26%	40, 10.36%	
EuroSCORE	6.06 ± 2.45	4.54 ± 2.08	< 0.001
Echocardiogram variables			
Preoperative LVEF (%)	30.36 ± 4.29	30.99 ± 3.87	0.124
Preoperative LVDd (cm)	7.11 ± 1.09	6.98 ± 1.05	0.258
Evaluated PAP (mmHg)	47.85 ± 18.11	48.24 ± 13.66	0.817
Previous Medical History (n,%)			
Acute Myocardial infarction	62, 50%	56, 14.51%	< 0.001
Atrial fibrillation	23, 18.55%	101, 26.17%	0.085
Diabetes Mellitus	15, 12.10%	34, 8.81%	0.280
Chronic Renal Failure	3, 2.42%	6, 1.55%	0.525
Hypertension	33, 26.61%	94, 24.35%	0.613
Liver Disease	4, 3.23%	14, 3.63%	0.833
COPD	12, 9.68%	8, 2.07%	< 0.001
Cancer	2, 1.61%	4, 1.04%	0.604
Smoking	42, 33.87%	57, 14.77%	< 0.001
Excessive alcohol	16, 12.90%	28, 7.25%	0.051
ACEI/ARB use	2, 1.61%	14, 3.63%	0.258
Ca^2+^-Blocker use	7, 5.64%	11, 2.85%	0.145
β-blocker use	0	7, 1.81%	0.130
Statin use	3, 2.42%	8, 2.07%	0.798
Diuretic use	9	25, 6.48%	0.799
Aspirin use	0	0	–
Clopidogrel use	0	7, 1.81%	0.130
Levosimendan use	0	0	–
Lyophilized Recombinant Human Brain Natriuretic	0	0	–

NYHA: New York Heart Association

LVEF: Left ventricular ejection fraction

COPD: Chronic obstructive pulmonary disease

PAP: Pulmonary artery pressure

LVDd: Left ventricular end-diastolic diameter

Mean ± SD

### Univariate analysis

Demographic and preoperative variables between survival group and non-survival group shown in Table 1. Age, gender, weight, NYHA class, EuroSCORE, AMI, chronic obstructive pulmonary disease (COPD) and smoking associated with survival in univariate analysis. Intraoperative and postoperative differences were shown in eTable 1 (online-only Data Supplement). These variables included type of cardiac surgery, CPB time, aortic cross clamp (ACC) time, intraoperative bleeding, postoperative CRRT, postoperative IABP and mechanical ventilation time. Patients who always used β-blockers during follow-up have been shown to be associated with survival in eTable 2 (online-only Data Supplement).

### Multivariate analysis

**Fig. 1:**
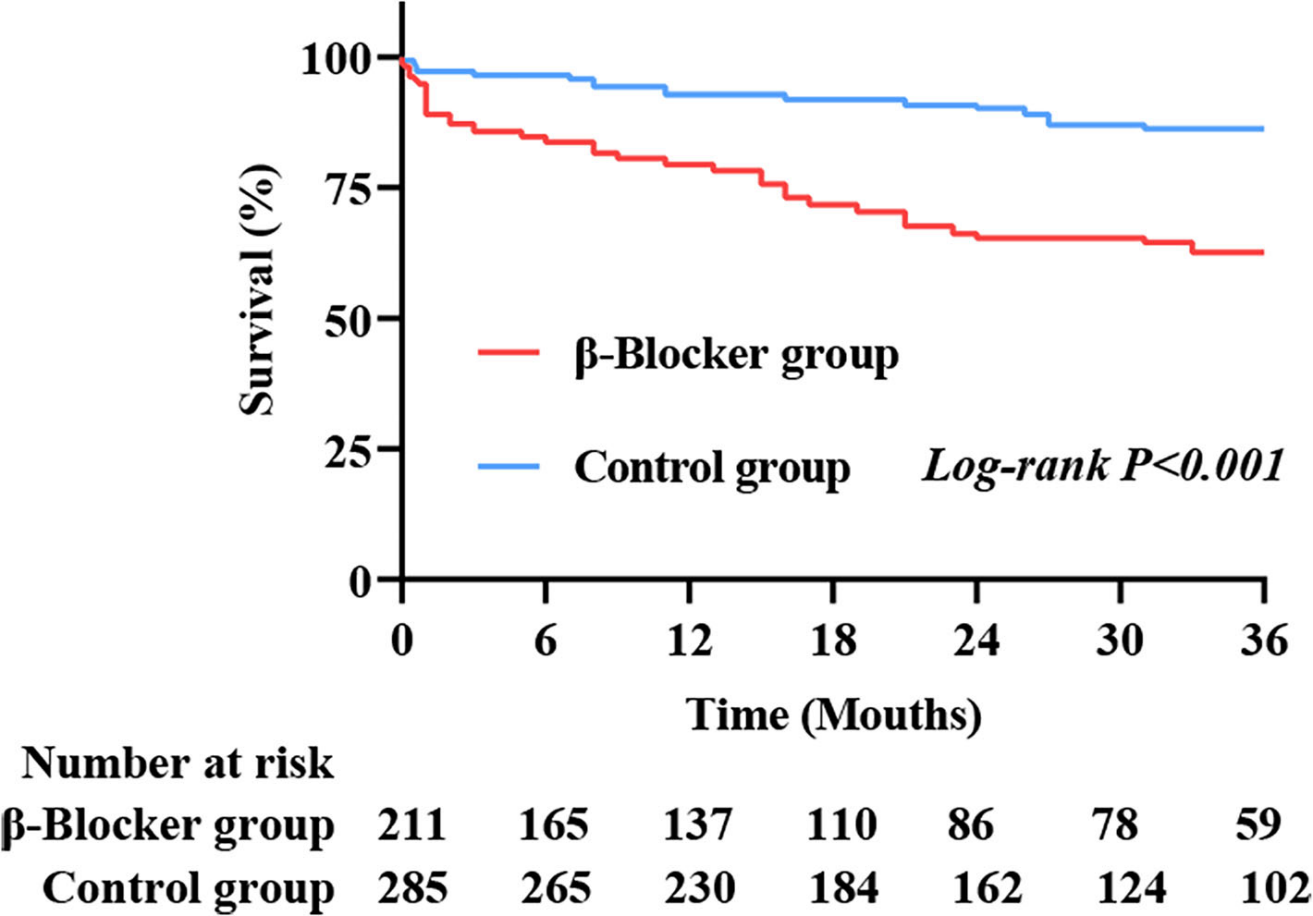
Kaplan Meier survival curves of the entire study cohort, with 95% confidence intervals (CI), showed that β-blocker was associated with a higher unadjusted mortality (86, 95%CI:82–90% vs. 62, 95%CI: 54–70%, *P* < 0.001). 14 patients who died suddenly after the operation were not included

In order to maintain the comparability between survival group and none-survival group, all covariates reaching a statistical significance (*P* ≤ 0.10) in univariate modeling were entered into a multivariable Cox regression model. In multivariable analysis, variables which associated with survival were shown as follows: weight > 70 kg (HR: 1.740, 95%CI: 1.048–2.889, *P* = 0.032), EuroSCORE > 6 (HR: 2.142, 95%CI: 1.225–3.743, *P* = 0.008), smoking (HR: 2.146, 95%CI: 1.034–4.451, *P* = 0.040), CPB > 216 min (HR: 8.004, 95%CI: 3.372–18.997, *P* < 0.001), postoperative IABP use (HR: 30.935, 95%CI: 10.328–92.661, P < 0.001), always use β-blocker during follow-up (HR: 2.056, 95%CI: 1.236–3.420, *P* = 0.005). Additionally, CABG + ventricular aneurysmectomy (HR: 5.683, 95%CI: 2.474–13.054, P < 0.001) and CABG + valvular surgery (HR: 4.314, 95%CI: 1.260–14.773, *P* = 0.020) increase long-term mortality, and MVR (HR: 0.169, 95%CI: 0.035–0.815, *P* = 0.027) decrease the mortality when CABG was regarded as reference. The detail variables of multivariable Cox analysis associated with survival were presented in Table 2. Kaplan Meier survival analysis of the entire study cohort, with 95% confidence intervals (CI), also showed that β-blocker was associated with a higher unadjusted mortality (Figs.1, 86, 95%CI:82–90% vs. 62, 95%CI: 54–70%, *P* < 0.001).

**Table 2:** Multivariate analysis of factors associated with survival

Variable	Hazard Ratio	95% Confidence Interval	P value
Age > 67 years old	0.645	0.343–1.214	0.174
Gender (male)	1.638	0.829–3.235	0.155
Weight > 70 kg	1.740	1.048–2.889	0.032
NYHA class			
I	Reference		
II	1.185	0.284–4.939	0.816
III	0.597	0.138–2.584	0.490
IV	0.973	0.186–5.085	0.974
EuroSCORE > 6	2.142	1.225–3.743	0.008
Preoperative LVEF > 33%	1.378	0.744–2.552	0.308
Preoperative acute myocardial infarction	0.713	0.192–2.641	0.612
Preoperative atrial fibrillation	1.399	0.763–2.566	0.278
Preoperative COPD	1.062	0.457–2.468	0.899
Smoking	2.146	1.034–4.451	0.040
Excessive alcohol	1.170	0.504–2.714	0.715
Type of cardiac surgery			
CABG	Reference		
AVR	0.307	0.043–2.202	0.240
MVR	0.169	0.035–0.815	0.027
AVR + MVR	0.284	0.063–1.273	0.100
David/Wheats/Bentall procedure	0.864	0.191–3.910	0.850
CABG +ventricular aneurysmectomy	5.683	2.474–13.054	< 0.001
CABG + valvular surgery	4.314	1.260–14.773	0.020
CPB > 216 min	8.004	3.372–18.997	< 0.001
ACC > 163 min	0.541	0.227–1.289	0.166
Intraoperative bleeding > 1.2 L	0.963	0.545–1.700	0.896
CRRT	1.190	0.445–3.186	0.729
Sepsis	0.742	0.198–2.785	0.659
IABP use	30.935	10.328–92.661	< 0.001
MV time > 23 h	0.985	0.576–1.684	0.956
Always use β-blocker	2.056	1.236–3.420	0.005

NYHA: New York Heart Association

CABG: Coronary artery bypass grafting

MVR: Mitral valve replacement/repair

ACC: Aortic Cross Clamp

IABP: Intra-aortic balloon pump

-LVEF: Left ventricular ejection fraction

AVR: Aortic valve replacement/repair

CPB: Cardiopulmonary bypass

CRRT: Continuous renal replacement therapy

MV: Mechanical ventilation

### Risk of long-term mortality with β-blockers in propensity-matched patients

**Fig. 2:**
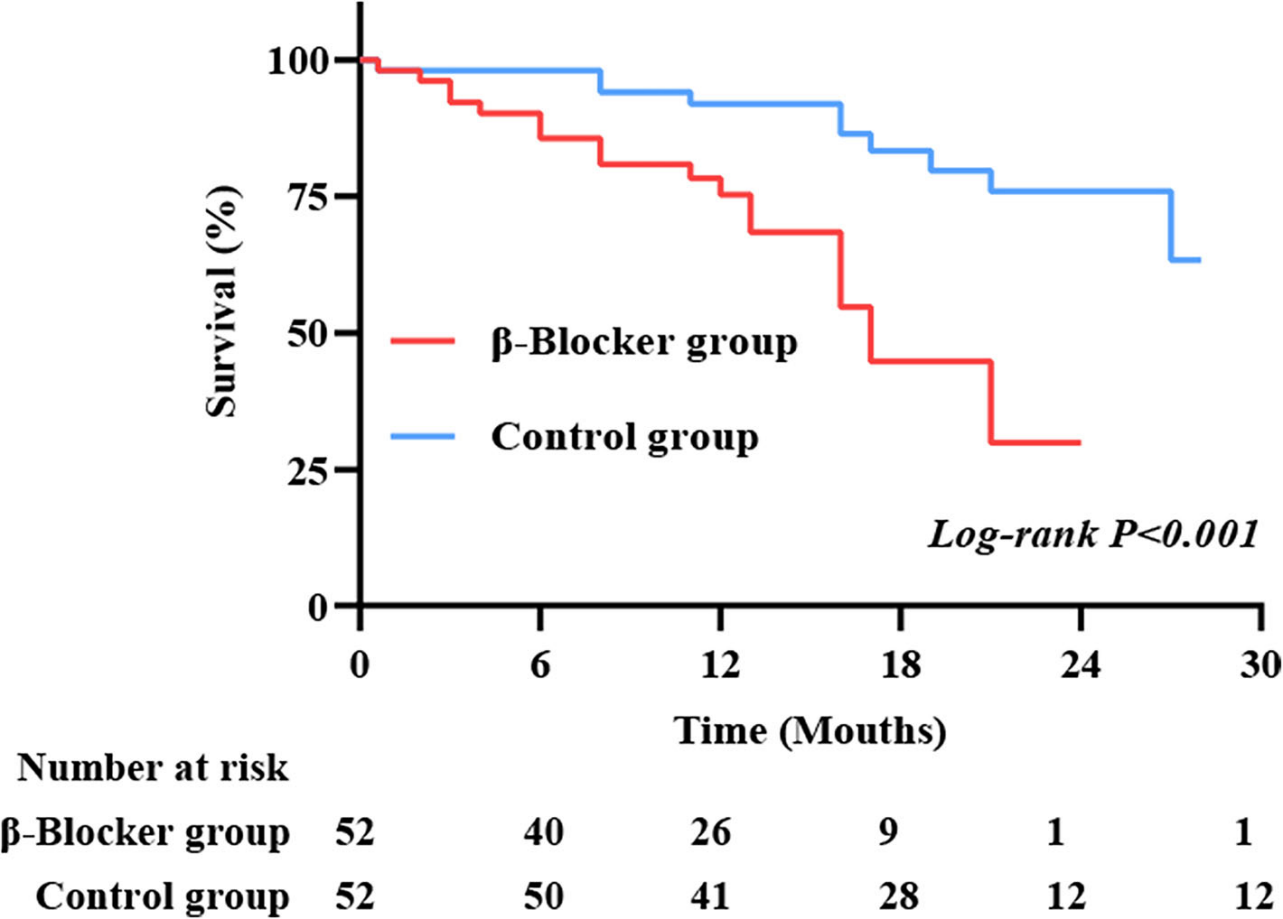
After matching, Kaplan-Meier survival curves showed that the survival rate was 24, 95%CI:0–48% in β-blocker group, while 74, 95%CI:58–90% in control group (P < 0.001)

The adjustment for selection bias was further addressed using a propensity score. Because β-blocker administration was identified as a novel risk factor of survival following cardiac surgery, we further studied this finding by pairing those who always used β-blockers with similar individuals who never or inconsistently used it. Before propensity-score matching, the absolute standardized differences of age, NYHA class, history of AMI, smoking, type of cardiac surgery, CPB time, intraoperative bleeding, drainage on the POD1, MV time, EuroSCORE and always use ACEI/ARB during follow up were − 14.6, − 2.2%, 14.9, 14.3, 26.8, 34.3, 12.0, 1.1, − 19.1%, 0.5 and 40.5% respectively. After matching, the absolute standardized differences of these 11 variables were 2.0, − 3.2%, 8.2, 3.5, − 8.8%, 21.2, 3.9, 3.9, 8.4, 22.1, 0.8 and 21.6% respectively. Therefore, these variables were well balance with a decreased standardized difference and had acceptable standardized mean differences (eFig. 1 in the online-only Data Supplement). Within the group of 211 individuals always used β-blockers, we could match 52 of them to 52 similar controls. After matching, there was no significant difference between propensity-matched groups with regard to baseline characteristics (eTable 3 in the online-only Data Supplement). In propensity-matched patients, β-Blocker group had higher rates of all-cause death (61.54% vs 80.77%, *P* = 0.030) and refractory heart failure (7.69% vs 32.69%, *P* = 0.003) compared with control group (Table 3). After matching, Kaplan-Meier survival curve showed that the survival rate was 24, 95%CI:0–48% in β-blocker group, while 74, 95%CI:58–90% in control group (Fig.2, *P* < 0.001).

**Table 3:** Association of β-blocker use at discharge and during follow-up with long-term outcomes in a matched cohort

Variables	Control (***N*** = 52)	Always users (N = 52)	Hazard ratio (95% CI)	P value
All-cause death (n, %)	32, 61.54%	42, 80.77%	2.08(1.18–3.66)	0.030
Refractory heart failure(n, %)	4, 7.69%	17, 32.69%	2.16(1.21–3.87)	0.003
Stroke (n, %)	13, 25%	9, 17.31%	0.63(0.31–1.30)	0.337
Refractory arrhythmia (n, %)	7, 13.46%	3, 5.77%	0.44(0.14–1.42)	0.183
Myocardial infarction (n, %)	8, 15.38%	9, 17.31%	1.41(0.68–2.91)	0.791
Renal failure (n, %)	0	3, 5.77%	4.55(1.39–14.94)	0.079
Respiratory failure (n, %)	0	1, 1.92%	6.36(0.84048.19)	0.315

In control group, patients had never used β-blocker (*n* = 0), or inconsistently used β-blocker (n = 52)

CI: Confidence Interval

In addition, stroke, refractory arrhythmia, myocardial infarction, renal failure and respiratory failure had no differences among two groups (Table 3). Furthermore, we further analyzed the data. The study population was divided into LVEF> 35% group and LVEF≤35% group according to their LVEF during follow-up (eTable 4 in the online-only Data Supplement). It showed that β-blocker had not only increased the mortality in pre-matched group (P < 0.001) but also increased mortality in matched group (*P* = 0.039) when LVEF was ≤35% during follow up. However, the β-blocker and control group had no differences in mortality when LVEF was > 35% during follow up (*P* = 0.406).

## Discussion

The data presented in this study demonstrate that patients with low LVEF (≤35%) undergoing a broad range of cardiac procedures have poor survival (24.31%). The risk factors for long-term survival were weight > 70 kg, EuroSCORE > 6, smoking, Cardiopulmonary bypass (CPB) > 216 min, postoperative Intra-aortic balloon pump (IABP) use, and always use β-blocker during follow-up. When CABG was regarded as reference, CABG + ventricular aneurysmectomy and CABG + valvular surgery are independent risk factors for long-term survival, and MVR is a preventive factor for long-term survival. Additionally, we found that β-blocker administration was associated with long-term mortality. To further assess the risk associated with β-blockers and adjust for potential biases, we paired individuals who always receive β-blockers to controls who inconsistently or never received β-blockers. On the basis of this propensity score, the Kaplan-Meier survival curves indicated that β-blocker administration was still associated with higher mortality during patient follow-up. Moreover, β-blocker could increase mortality when postoperative heart function was not improved; and might not increase mortality when postoperative heart function had been improved.

To our knowledge, this is the first study to show a relationship between β-blocker therapy after hospital discharge and increased risk for long-term mortality. It may offer new insights into the association between β-blocker therapy and long-term survival. Current evidence regarding the beneficial effect of β-blocker may be conclusive, especially in CABG patients [26–28]. In a long-term observational study of 5926 consecutive patient undergoing isolated CABG, Zhang H et al. reported that consistent use of β-blocker after hospital discharger was associated with a lower risk of death and composite cardiovascular events [26]. In a prospective cohort of 3102 patients, Chan AY et al. reported that Patients discharged with β-blockers after cardiac surgery exhibit a substantially lower mortality rate during long-term follow-up [29]. In our study, half of the patients in the non-survival group are accompanied with AMI, while 56/386 in the survival group (*P* < 0.001), and all patients in current study received selective CABG. According to these previous studies, the β-blocker is routinely recommended to be a discharge prescription [26, 29]. However, we found that if heart function does not improve after surgery, β-blockers might increase mortality. For patients with significantly improved heart function after surgery, β-blockers might not increase mortality.

Several differences should be highly emphasized. Firstly, these previous studies contained a larger proportion of LVEF ≥50%. In our study the mean LVEF is 30.83% (standard deviation: 3.97%). In other words, the previous condition of patients in our study were worse than those in previous studies. Secondly, those previous studies had a large population of isolated CABG (> 60%). There were 17.45% patients who underwent isolated CABG in our study. It indicated that our study had broad range of cardiac procedures. Finally, we considered that all patients had received selective operation in our study, whereas there were 58.48% patients who underwent urgent or emergent operation in Dr. Chan AY’ s study [29]. Patients who were discharged with β-blocker after urgent cardiac surgery may have a lower rate of death [30]. That may be the reason why their β-blocker group showed positive outcomes. Therefore, our findings reduce the evidence supporting the use of discharge β-blocker after cardiac surgery. In addition, we have addressed criticisms that the β-blockers reduce mortality in patients after various kinds of cardiac procedures [29]. A potential mechanistic explanation for our findings arose from a pharmacological analysis that demonstrated that β-blocker acted as a negative inotropic agent might further decrease the cardiac output when the cardiac function had irreversibly impaired. That is the reason why β-blocker might increase long-term mortality when heart function could not be improved during follow-up.

Our study also indicated that many factors on multivariable analysis were association with long-term survival. These risk factors including weight, EuroSCORE, smoking, CPB and postoperative IABP utilization. In this sense, our experience may add new clues to be implanted into clinical practice. A previous study have shown that IABP improved the outcomes in high risk patients undergoing CABG [31]. In our study, patients who postoperatively initiated IABP were implemented CABG+ ventricular aneurysmectomy (*n* = 4) and CABG + valvular surgery (*n* = 8). When CABG was regarded as reference, CABG + ventricular aneurysmectomy and CABG + valvular surgery are independent risk factors for long-term survival. Therefore, the use of postoperative IABP is a marker of severity illness rather than primary cause of a worse outcome. Finally, our study demonstrated a similar conclusion that MVR would improve survival even in patients with low LVEF [3]. It may be necessary that those patients with low LVEF should undergo MVR as soon as possible.

Limitations of the present study also need to be acknowledged. Our study design involved one center’s experiences with the inherent disadvantages of a retrospective study, which is highly prone to bias. This observational study could have been influenced by potential biases. It should be indicated that our patient population might be very heterogenous, with many procedures of varying complexity pooled together. We used propensity score matching to avoid these biases. The type of cardiac surgery was regarded as one of the covariates for our propensity score matching. After the matching, the standard mean difference (SMD) of the surgical procedures of the two groups decreased from 26.8 to 8.8%. Meanwhile, the *P* value of procedures was 0.438 (eTable 3) after propensity matching (eTable 1, the pre-matching *P* = 0.005). We had tried our best to avoid the bias. However, with this analysis, we removed a large number of patients from the analysis but may have elevated the statistical errors. Furthermore, in the early period from January 2007 to December 2012, 155 patients (155/230, 67.4%) were lost to follow-up. However, there were 19 patients (19/454, 4.2%) who were lost to follow-up from January 2013 to September 2019. We did not exclude patients from January 2007 to December 2012. Because the data(2007–2013) is still valuable, important and useful to be shared when the final results are not changed (eTable 5 in the online-only Data Supplement). However, 174 patients were lost to follow-up. This accounts for as much as 25% of the whole patient population and can essentially confound the results. Moreover, we could not collect detailed data about β-blocker therapy during follow up, such as daily heart rate, timing of changed dosage and reasons of changed dosage. Finally, there were 285 patients who did not re-examine echocardiography and/or magnetic resonance imaging (MRI) after 1-year of hospital discharge in this study cohort. Therefore, we could not collect echocardiographic and/or MRI data in these patients. These factors that affect assignment to treatment and outcomes but cannot be observed are hidden bias in our study. Any hidden bias due to latent variables might have remained after matching, which could have led to some statistical errors. Thus, we recommend further high-quality trials to answer questions about the mechanisms of action, effectiveness on subgroups, dose-response, length of therapy, functional outcome, and quality of life after β-blocker use for patients with low LVEF.

## Conclusions

The risk factors for long-term survival were weight > 70 kg, EuroSCORE > 6, smoking, CPB > 216 min, postoperative IABP use, and always use β-blocker during follow-up. Meanwhile, β-blocker might not increase mortality when heart function had been improved after cardiac surgery. The discharge prescription of β-blocker should be cautiously administrated in those patients.

## Supplementary information

**Additional file 1: eFig. 1.** The standardized mean difference was visually presented.

**Additional file 2: eTable 1.** Intraoperative and postoperative variables.

**Additional file 3: eTable 2.** Follow-up variables.

**Additional file 4: eTable 3.** Baseline Demographic and clinical characteristics in propensity-matched cohort.

**Additional file 5: eTable 4.** the long-term mortality after surgery in different groups.

**Additional file 6: eTable 5.** Multivariate analysis of factors related to survival of patients from January 1, 2013 to September 1, 2019.

## Data Availability

The datasets generated and/or analyzed during the current study are not publicly available [some patients did not allow us to publish their medical records] but are available from the corresponding author upon reasonable request.

## Abbreviations

CPB: Cardiopulmonary bypass
LVEF: Left ventricular ejection fraction
IABP: Intra-aortic balloon pump
CABG: Coronary artery bypass grafting
AVR: Aortic valve replacement or repair
MVR: Mitral valve replacement or repair
NYHA: New York heart association
AMI: Acute myocardial infarction
POD1: The first postoperative day
MV: Mechanical ventilation
