# Supplementary figures and images for: Evaluation of β-blocker therapy for long-term outcomes in patients with low ejection fraction after cardiac surgery
Source: BMC Cardiovasc Disord. 2020 Aug 20;20:379. doi: 10.1186/s12872-020-01651-6 (PMC7439680; doi:10.1186/s12872-020-01651-6)

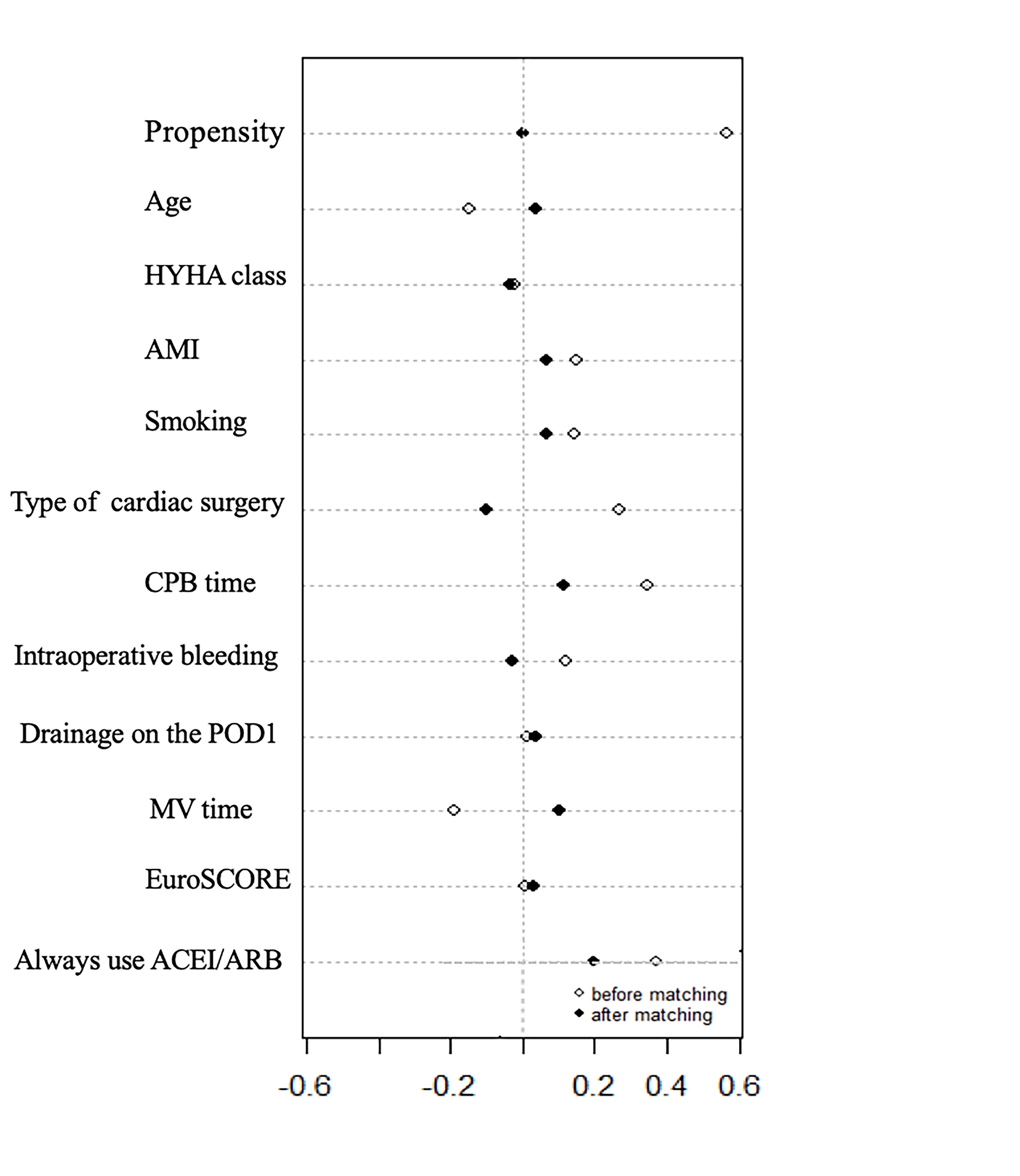

Supplement: Supplementary file 1 — Additional file 1: eFig. 1. The standardized mean difference was visually presented. [file 12872_2020_1651_MOESM1_ESM.tif]
