# Supplementary material for: Evaluation of β-blocker therapy for long-term outcomes in patients with low ejection fraction after cardiac surgery
Source: BMC Cardiovasc Disord. 2020 Aug 20;20:379. doi: 10.1186/s12872-020-01651-6 (PMC7439680; doi:10.1186/s12872-020-01651-6)
Supplement: Supplementary file 2 — Additional file 2: eTable 1. Intraoperative and postoperative variables. [file 12872_2020_1651_MOESM2_ESM.docx]

| **eTable 1. Intraoperative and postoperative variables** | | | | | | |
| --- | --- | --- | --- | --- | --- | --- |
| **Variable** | | **Non-Survival**  **(n=124)** | | **Survival**  **(n=386)** | | **P value** |
| **Type of cardiac surgery (n, %)** | |  | |  | | 0.005 |
| CABG | | 38, 30.64% | | 51, 13.21% | |  |
| AVR | | 2, 1.61% | | 19, 4.92% | |  |
| MVR | | 14, 11.29% | | 168, 43.52% | |  |
| AVR + MVR | | 19, 15.32% | | 101, 26.17% | |  |
| David/Wheats/Bentall procedure | | 22, 17.75% | | 44, 11.40% | |  |
| CABG +ventricular aneurysmectomy | | 17, 13.71% | | 0 | |  |
| CABG + valvular surgery | | 12, 9.68% | | 3, 0.78% | |  |
| Atrial fibrillation ablation (n, %) | | 16, 12.90% | | 65, 16.84% | | 0.297 |
| CPB (minutes) | | 228.36±65.49 | | 165.32±54.50 | | <0.001 |
| ACC (minutes) | | 160.12±50.80 | | 120.83±48.06 | | <0.001 |
| DHCA (n, %) | | 2, 1.61% | | 4, 1.04% | | 0.604 |
| Intraoperative bleeding (L) | | 1.19±0.87 | | 0.93±0.58 | | 0.002 |
| **Postoperative outcomes** | |  | |  | |  |
| Drainage on the POD1 (ml) | | 45 (0-702) | | 0 (0-408) | | 0.195 |
| Re-operation (n, %) | | 7, 5.64% | | 14, 3.63% | | 0.224 |
| CRRT (n, %) | | 10, 8.06% | | 9, 2.33% | | 0.003 |
| Sepsis (n, %) | | 4, 3.23% | | 4, 1.04% | | 0.056 |
| Septic shock (n, %) | | 0 | | 0 | | — |
| ARDS (n, %) | | 0 | | 0 | | — |
| Brain injury (n, %) | | 1, 0.81% | | 3, 0.78% | | 0.974 |
| ECMO use (n, %) | | 0 | | 0 | | — |
| IABP use (n, %) | | 7, 5.64% | | 5, 1.29% | | 0.005 |
| Death in ICU | | 29, 23.39% | | 0 | | <0.001 |
| Mechanical ventilation time (hours) | | 18(13-42) | | 17(13-21) | | 0.020 |
| ICU stay time | | 4.55±2.57 | | 4.20±2.46 | | 0.194 |
| CABG: Coronary artery bypass grafting | AVR: Aortic valve replacement/repair | | | | MVR: Mitral valve replacement/repair | |
| CPB: Cardiopulmonary bypass | ACC: Aortic Cross Clamp | | | | Mean ± SD / Median (interquartile range) | |
| DHCA: Deep hypothermic circulatory arrest | | | CRRT: Continuous renal replacement therapy | | | |
| ARDS: Acute Respiratory Distress Syndrome  IABP: Intra-aortic balloon pump  POD1:The first postoperative day | | | ECMO: Extracorporeal membrane oxygenation  ICU: Intensive care unit | | | |
