# Supplementary material for: Evaluation of β-blocker therapy for long-term outcomes in patients with low ejection fraction after cardiac surgery
Source: BMC Cardiovasc Disord. 2020 Aug 20;20:379. doi: 10.1186/s12872-020-01651-6 (PMC7439680; doi:10.1186/s12872-020-01651-6)
Supplement: Supplementary file 3 — Additional file 3: eTable 2. Follow-up variables. [file 12872_2020_1651_MOESM3_ESM.docx]

| **eTable 2. Follow-up variables** | | | | |
| --- | --- | --- | --- | --- |
| **Variable** | **Non-Survival**  **(n=110)** | | **Survival**  **(n=386)** | **P value** |
| **ECHO before leaving the hospital** |  | |  |  |
| LVEF (%) | 34.03±4.97 | | 34.30±4.87 | 0.607 |
| LVDd (cm) | 6.64±0.88 | | 6.51±0.86 | 0.165 |
| Aortic valve regurgitation (n, %) |  | |  | 0.177 |
| None | 41, 37.27% | | 119, 30.83% |  |
| Mild | 63, 57.27% | | 240, 62.18% |  |
| Moderate | 6, 5.45% | | 27, 6.99% |  |
| Sever | 0 | | 0 |  |
| Mitral valve regurgitation (n, %) |  | |  | 0.379 |
| None | 24, 21.82% | | 98, 25.39% |  |
| Mild | 70, 63.64% | | 241, 62.43% |  |
| Moderate | 16, 14.54% | | 47, 12.18% |  |
| Sever | 0 | | 0 |  |
| Tricuspid valve regurgitation (n, %) |  | |  | 0.318 |
| None | 5, 4.54% | | 36, 9.33% |  |
| Mild | 81, 73.64% | | 271, 70.21% |  |
| Moderate | 22, 20% | | 72, 18.65% |  |
| Sever | 2, 1.82% | | 7, 1.81% |  |
| **Oral drugs during follow-up (n, %)** |  | |  |  |
| Always use ACEI/ARB | 39, 35.45% | | 116, 30.05% | 0.316 |
| Always use β-blocker | 76, 69.09% | | 135, 34.97% | <0.001 |
| Always use aspirin | 53, 48.18% | | 205, 53.11% | 0.362 |
| Always use clopidogrel | 37, 33.64% | | 161, 41.71% | 0.149 |
| Always use diuretics | 27, 24.54% | | 86, 22.28% | 0.575 |
| Digoxin / Cedilanid uses | 25, 22.73% | | 65, 16.84% | 0.142 |
| **Events during follow up (n, %)** |  | |  |  |
| Heart transplantation | 7, 6.36% | | 26, 6.75% | 0.885 |
| Atrial fibrillation | 28, 25.45% | | 75, 19.43% | 0.447 |
| Ventricular arrhythmia | 7, 6.36% | | 36, 9.33% | 0.199 |
| Other arrhythmia | 42, 38.18% | | 108, 27.98% | 0.210 |
| ECHO: Echocardiography | | LVEF: Left ventricular ejection fraction | | |
| LVDd: Left ventricular end-diastolic diameter  ARB: Angiotensin Receptor Blockers | | ACEI: Angiotensin-Converting Enzyme Inhibitors | | |

There 14 patients who had no ECHO variables in non-survival group. They suddenly died after surgery.
