# Supplementary material for: Evaluation of β-blocker therapy for long-term outcomes in patients with low ejection fraction after cardiac surgery
Source: BMC Cardiovasc Disord. 2020 Aug 20;20:379. doi: 10.1186/s12872-020-01651-6 (PMC7439680; doi:10.1186/s12872-020-01651-6)
Supplement: Supplementary file 4 — Additional file 4: eTable 3. Baseline Demographic and clinical characteristics in propensity-matched cohort. [file 12872_2020_1651_MOESM4_ESM.docx]

| **eTable 3: Baseline Demographic and clinical characteristics in propensity-matched cohort** | | | | | | | | |
| --- | --- | --- | --- | --- | --- | --- | --- | --- |
| **Variable** | | **Entire cohort prior matching** | | | | **Propensity-matched cohort** | | |
|  | **Control***  **(n=285)** | | **Always users (n=211)** | | **P value** | **Control****  **(n=52)** | **Always users**  **(n=52)** | **P value** |
| Age (year) | 60.02±12.24 | | 58.08±11.22 | | 0.071 | 60.42±14.10 | 58.69±9.23 | 0.461 |
| Gender (male, %) | 188, 65.96% | | 140, 66.35% | | 0.928 | 38, 73.08% | 34, 65.38% | 0.395 |
| Weight (kg) | 63.30±11.22 | | 64.26±12.88 | | 0.379 | 65.13±12.28 | 64.10±11.34 | 0.658 |
| NYHA class (n, %) |  | |  | | 0.001 |  |  | 0.455 |
| I | 7, 2.46% | | 5, 2.37% | |  | 3, 5.77% | 2, 3.85% |  |
| II | 87, 30.53% | | 72, 34.12% | |  | 29, 55.77% | 27, 51.92% |  |
| III | 163, 57.19% | | 113, 53.56% | |  | 16, 30.77% | 17, 32.69% |  |
| IV | 28, 9.82% | | 21, 9.95% | |  | 4, 7.69% | 6, 11.54% |  |
| EuroSCORE | 4.90±2.32 | | 4.84±2.16 | | 0.790 | 4.71±1.83 | 4.77±1.8 | 0.871 |
| **Echocardiogram variables** |  | |  | |  |  |  |  |
| Preoperative LVEF (%) | 30.78±4.06 | | 30.90±3.80 | | 0.753 | 31.75±5.65 | 31.72±3.91 | 0.968 |
| Preoperative LVDd (cm) | 7.03±1.07 | | 6.97±1.01 | | 0.501 | 6.92±1.08 | 7.01±1.12 | 0.685 |
| Evaluated PAP (mmHg) | 47.65±14.48 | | 48.75±15.04 | | 0.442 | 42 (33-52) | 44 (32-66) | 0.432 |
| **Previous Medical History (n,%)** |  | |  | |  |  |  |  |
| Acute Myocardial infarction | 53, 18.60% | | 57, 27.01% | | 0.026 | 14, 26.92% | 11, 21.15% | 0.491 |
| Atrial fibrillation | 65, 22.81% | | 59, 27.96% | | 0.190 | 14, 26.92% | 11, 21.15% | 0.491 |
| Diabetes Mellitus | 27, 9.47% | | 18, 8.53% | | 0.718 | 6, 11.54% | 4, 7.69% | 0.506 |
| Chronic Renal Failure | 4, 1.40% | | 3, 1.42% | | 0.986 | 0 | 0 | — |
| Hypertension | 72, 25.26% | | 51, 24.17% | | 0.781 | 11, 21.15% | 12, 23.08% | 0.813 |
| Liver Disease | 13, 4.56% | | 3, 1.42% | | 0.500 | 3, 5.77% | 1, 1.92% | 0.308 |
| COPD | 10, 3.51% | | 8, 3.79% | | 0.868 | 2, 3.85% | 1, 1.92% | 0.558 |
| Cancer | 4, 1.40% | | 1, 0.47% | | 0.306 | 0 | 0 | — |
| Smoking | 46, 16.14% | | 48, 22.75% | | 0.063 | 12, 23.08% | 15, 28.85% | 0.502 |
| Excessive alcohol | 20, 7.02% | | 23, 10.90% | | 0.129 | 6, 11.54% | 5, 9.61% | 0.750 |
| ACEI/ARB use | 10, 3.51% | | 6, 2.84% | | 0.675 | 1, 1.92% | 0 | 0.315 |
| Ca^2+^-Blocker use | 10, 3.51% | | 8, 3.79% | | 0.880 | 0 | 0 | — |
| β-blocker use | 4, 1.40% | | 3, 1.42% | | 0.994 | 0 | 0 | — |
| Statin use | 4, 1.40% | | 7, 3.32% | | 0.154 | 0 | 0 | — |
| Diuretic use | 17, 5.96% | | 17 ,8.06% | | 0.375 | 4, 7.69% | 2, 3.85% | 0.428 |
| Aspirin use | 0 | | 0 | | — | 0 | 0 | — |
| Clopidogrel use | 6, 2.11% | | 1, 0.47% | | 0.126 | 0 | 0 | — |
| Levosimendan use | 0 | | 0 | | — | 0 | 0 | — |
| Lyophilized Recombinant Human Brain Natriuretic | 0 | | 0 | | — | 0 | 0 | — |
| **Type of cardiac surgery (n, %)** |  | |  | | 0.005 |  |  | 0.438 |
| CABG | 48, 16.84% | | 36, 17.06% | |  | 12, 23.08% | 11, 21.15% |  |
| AVR | 14, 4.91% | | 7, 3.32% | |  | 3, 5.77% | 5, 9.61% |  |
| MVR | 116, 40.70 | | 64, 30.33% | |  | 17, 32.69% | 16, 30.77% |  |
| AVR + MVR | 73, 25.61% | | 45 ,21.33% | |  | 11, 21.15% | 12, 23.08% |  |
| David/Wheats/Bentall procedure | 29, 10.18% | | 33, 15.64% | |  | 6, 11.54% | 8, 15.38% |  |
| CABG +ventricular aneurysmectomy | 3, 1.05% | | 13, 6.16% | |  | 2, 3.85% | 0 |  |
| CABG + valvular surgery | 2, 0.70% | | 13, 6.16% | |  | 1, 1.92% | 0 |  |
| Atrial fibrillation ablation (n, %) | 46, 16.14% | | 35, 16.59% | | 0.894 | 9, 17.31% | 5, 9.61% | 0.250 |
| CPB (minutes) | 170.21±61.59 | | 192.67±59.74 | | <0.001 | 203.56±73.19 | 186.60±59.02 | 0.196 |
| ACC (minutes) | 121.97±51.13 | | 142.09±50.07 | | <0.001 | 152.23±62.74 | 134.58±54.49 | 0.129 |
| DHCA (n, %) | 2, 0.70% | | 4, 1.90% | | 0.229 | 1, 1.92% | 3, 5.77% | 0.308 |
| Intraoperative bleeding (L) | 0.92±0.48 | | 1.04±0.72 | | 0.021 | 0.94±0.66 | 1.01±0.86 | 0.616 |
| **Postoperative outcomes** |  | |  | |  |  |  |  |
| Drainage on the POD1 (ml) | 45 (0-702) | | 0 (0-407) | | 0.001 | 105(0-630) | 110(0-390) | 0.640 |
| Re-operation (n, %) | 12, 4.21% | | 9, 4.27% | | 0.956 | 5, 9.61% | 3, 5.77% | 0.462 |
| CRRT (n, %) | 6, 2.11% | | 10, 4.74% | | 0.102 | 2, 3.85% | 3, 5.77% | 0.647 |
| Sepsis (n, %) | 5, 1.75% | | 3, 1.42% | | 0.775 | 0 | 1, 1.92% | 0.315 |
| Septic shock (n, %) | 0 | | 0 | | — | 0 | 0 | — |
| ARDS (n, %) | 0 | | 0 | | — | 0 | 0 | — |
| Brain injury (n, %) | 1, 0.35% | | 2, 0.95% | | 0.397 | 0 | 0 | — |
| ECMO use (n, %) | 0 | | 0 | | — | 0 | 0 | — |
| IABP use (n, %) | 8, 2.81% | | 3, 1.42% | | 0.300 | 0 | 0 | — |
| Death in ICU | 7, 2.46% | | 9, 4.27% | | 0.260 | 0 | 0 | — |
| MV time (hours) | 18 (13-43) | | 17 (13-21) | | 0.020 | 18 (13-24) | 17 (11-21) | 0.210 |
| ICU stay time | 4.40±2.68 | | 4.16±2.19 | | 0.300 | 4 (2-6) | 4 (2-5) | 0.527 |
| **ECG before leaving the hospital** |  | |  | |  |  |  |  |
| LVEF (%) | 34.03±5.05 | | 34.67±4.51 | | 0.148 | 34.04±4.33 | 34.84±4.55 | 0.360 |
| LVDd (cm) | 6.55±0.92 | | 6.50±0.78 | | 0.460 | 6.63±1.01 | 6.49±0.81 | 0.441 |
| Aortic valve regurgitation (n, %) |  | |  | | 0.177 |  |  | — |
| None | 79, 27.72% | | 84, 39.81% | |  | 25, 48.08% | 25, 48.08% |  |
| Mild | 188, 65.96% | | 113, 53.55% | |  | 23, 44.23% | 23, 44.23% |  |
| Moderate | 18, 6.32% | | 14, 6.64% | |  | 4, 7.69% | 4, 7.69% |  |
| Sever | 0 | | 0 | |  | 0 | 0 |  |
| Mitral valve regurgitation (n, %) |  | |  | | 0.379 |  |  | 0.609 |
| None | 66, 23.16% | | 60, 28.44% | |  | 9, 17.31% | 9, 17.31% |  |
| Mild | 175, 61.40% | | 133, 63.03% | |  | 34, 65.38% | 37, 71.15% |  |
| Moderate | 44, 15.44% | | 18, 8.53% | |  | 9, 17.31% | 6, 11.54% |  |
| Sever | 0 | | 0 | |  | 0 | 0 |  |
| Tricuspid valve regurgitation (n, %) |  | |  | | 0.318 |  |  | 0.848 |
| None | 22, 7.72% | | 24, 11.38% | |  | 3, 5.77% | 3, 5.77% |  |
| Mild | 199, 69.83% | | 151, 71.56% | |  | 35, 67.31% | 34, 65.38% |  |
| Moderate | 60, 21.05% | | 31, 14.69% | |  | 14, 26.92% | 15, 28.85% |  |
| Sever | 4, 1.40% | | 5, 2.37% | |  | 0 | 0 |  |
| **Oral drugs during follow-up (n, %)** |  | |  | |  |  |  |  |
| Always use ACEI/ARB | 18, 6.32% | | 137, 64.93% | | <0.001 | 17, 32.69% | 20, 38.46% | 0.539 |
| Always use aspirin | 137, 48.04% | | 129, 61.14% | | 0.046 | 24, 46.15% | 21, 40.38% | 0.553 |
| Always use clopidogrel | 103, 36.14% | | 94, 44.55% | | 0.053 | 16, 30.77% | 16, 30.77% | — |
| Always use diuretics | 103,36.14% | | 94, 44.55% | | 0.053 | 13, 25% | 12, 23.08% | 0.819 |
| Digoxin / Cedilanid uses | 57, 20.00% | | 32, 15.17% | | 0.173 | 9, 17.31% | 9, 17.31% | — |
| **Events during follow up (n, %)** |  | |  | |  |  |  |  |
| Heart transplantation | 15, 5.26% | | 18, 8.53% | | 0.145 | 1, 1.92% | 2, 3.85% | 0.558 |
| Atrial fibrillation | 53, 18.60% | | 48, 22.75% | | 0.256 | 14, 26.92% | 11, 21.15% | 0.491 |
| Ventricular arrhythmia | 24, 8.42% | | 19, 9.00% | | 0.819 | 1, 1.92% | 3, 5.77% | 0.308 |
| Other arrhythmia | 89, 31.23% | | 54, 25.59% | | 0.171 | 20, 38.46% | 15, 28.85% | 0.299 |
| NYHA: New York Heart Association  LVEF: Left ventricular ejection fraction  LVDd: Left ventricular end-diastolic diameter  AVR: Aortic valve replacement/repair  CPB: Cardiopulmonary bypass  DHCA: Deep hypothermic circulatory arrest  ARDS: Acute Respiratory Distress Syndrome  IABP: Intra-aortic balloon pump  ECG: Echocardiography  ARB: Angiotensin Receptor Blockers | | | | COPD: Chronic obstructive pulmonary disease  PAP: Pulmonary artery pressure  CABG: Coronary artery bypass grafting  MVR: Mitral valve replacement/repair  ACC: Aortic Cross Clamp  CRRT: Continuous renal replacement therapy  ECMO: Extracorporeal membrane oxygenation  ICU: Intensive care unit  ACEI: Angiotensin-Converting Enzyme Inhibitor | | | | |

*: In control group, patients had never used β-blocker (n=24), or inconsistently used β-blocker (n=261).

**: In control group, patients had never used β-blocker (n=0), or inconsistently used β-blocker (n=52).
