# Supplementary material for: Evaluation of β-blocker therapy for long-term outcomes in patients with low ejection fraction after cardiac surgery
Source: BMC Cardiovasc Disord. 2020 Aug 20;20:379. doi: 10.1186/s12872-020-01651-6 (PMC7439680; doi:10.1186/s12872-020-01651-6)
Supplement: Supplementary file 5 — Additional file 5: eTable 4. the long-term mortality after surgery in different groups. [file 12872_2020_1651_MOESM5_ESM.docx]

| **eTable 4: the long-term mortality after surgery in different groups** | | | | |
| --- | --- | --- | --- | --- |
|  | | **Always user group** | **Control group** | **P value** |
| **Unmatched**  **Cohort*** | **LVEF>35%** | 29/84, 34.5% | 11/96, 11.5%% | <0.001 |
|  | **LVEF≤35%** | 44/124, 35.5% | 23/182, 12.6% | <0.001 |
| **Matched**  **Cohort** | **LVEF>35%** | 5/11, 45.5% | 18/30, 60.0% | 0.406 |
|  | **LVEF≤35%** | 5/19, 26.3% | 24/44, 54.5% | 0.039 |

*there were 14 patients who quickly died in postoperative days. And there were 10 patients who died in 3 months after discharge from hospital. Therefore, they did not had postoperative echocardiogram data.
