# Supplementary material for: Evaluation of β-blocker therapy for long-term outcomes in patients with low ejection fraction after cardiac surgery
Source: BMC Cardiovasc Disord. 2020 Aug 20;20:379. doi: 10.1186/s12872-020-01651-6 (PMC7439680; doi:10.1186/s12872-020-01651-6)
Supplement: Supplementary file 6 — Additional file 6: eTable 5. Multivariate analysis of factors related to survival of patients from January 1, 2013 to September 1, 2019. [file 12872_2020_1651_MOESM6_ESM.docx]

| **eTable 5: Multivariate analysis of factors related to survival of patients from January 1, 2013 to September 1, 2019** | | | |
| --- | --- | --- | --- |
| **Variable** | **Hazard Ratio** | **95% Confidence Interval** | **P value** |
| Weight > 70 kg | 1.365 | 0.880-2.117 | 0.165 |
| EuroSCORE > 6 | 1.913 | 1.264-2.897 | 0.002 |
| Smoking | 2.183 | 1.393-3.421 | 0.001 |
| CPB > 216 minutes | 4.009 | 2.551-6.298 | 0.000 |
| IABP use | 9.739 | 3.998-23.720 | 0.000 |
| Always use β-blocker | 2.574 | 1.654-4.006 | 0.000 |
| CABG: Coronary artery bypass grafting; IABP: Intra-aortic balloon pump; CPB: Cardiopulmonary bypass | | | |
